# Knockdown of *BmTCP-1β* Delays BmNPV Infection *in vitro*

**DOI:** 10.3389/fmicb.2019.00578

**Published:** 2019-03-22

**Authors:** Xue-yang Wang, Zuo-min Shao, Qian-ying Chen, Jia-ping Xu, Xia Sun, Zhen-ping Xu, Mu-wang Li, Yang-chun Wu

**Affiliations:** ^1^Jiangsu Key Laboratory of Sericultural Biology and Biotechnology, School of Biotechnology, Jiangsu University of Science and Technology, Zhenjiang, China; ^2^The Key Laboratory of Silkworm and Mulberry Genetic Improvement, Ministry of Agriculture, Sericultural Research Institute, Chinese Academy of Agricultural Sciences, Zhenjiang, China; ^3^School of Life Sciences, Anhui Agricultural University, Hefei, China

**Keywords:** *Bombyx mori*, BmNPV, chaperonin containing t-complex polypeptide 1β, protein folding, response mechanism

## Abstract

The molecular mechanism of silkworm resistance to *Bombyx mori* nucleopolyhedrovirus (BmNPV) infection remains unclear. The chaperonin containing t-complex polypeptide 1 (TCP-1) is essential for the folding of tubulin and actin to produce stable and functional competent protein conformation. However, little is known about this protein in silkworm. In the present study, a gene encoding the TCP-1β protein in silkworm was characterized, which has an open reading fragment of 1,611 bp encoding a predicted 536 amino acid residue-protein with a molecular weight of approximately 57.6 kDa containing a Cpn60_TCP1 functional domain. The sequence conservation is 81.52%. The highest level of *BmTCP-1β* mRNA expression was found in the midgut, while the lowest was in the hemolymph. To further study the function of *BmTCP-1β*, expression was knocked down with siRNA *in vitro*, resulting in significant downregulation of the selected cytoskeletal-related genes, *actin* and *tubulin*, which was also confirmed by overexpression of *BmTCP-1β* in BmN cells using the pIZT/V5-His-mCherry insect vector. Moreover, knockdown of *BmTCP-1β* significantly prolonged the infection process of BmNPV in BmN cells, which was also verified by overexpression of *BmTCP-1β* in BmN cells. Based on the results of the present study, we concluded that *BmTCP-1β* plays a vital role in BmNPV infection by regulating the expression of *tubulin* and *actin*. Taken together, our work provides valuable data for the clarification of the molecular mechanism of silkworm resistance to BmNPV infection.

## Introduction

The silkworm *Bombyx mori* L. (Lepidoptera: Bombycidae) has been domesticated for more than 5000 years and still plays an important role in many developing countries. Moreover, *B. mori* is a good model organism for the study of insect genetics and immunology (Goldsmith et al., 2005; Shao et al., 2012). *B. mori* nucleopolyhedrovirus (BmNPV) is a primary silkworm pathogen that causes serious economic losses annually. Interestingly, certain silkworm strains exhibit high resistance to BmNPV infection (Bao et al., 2009); however, the molecular underlying mechanism has not yet been fully elucidated.

The chaperonins are key molecular complexes that ensure the correct folding of proteins to produce energetically stable and functionally competent protein conformations (Coghlin et al., 2006). The eukaryotic TRiC (TCP-1 ring complex, also called CCT, chaperonin-containing TCP-1) is composed of two back-to-back stacked rings with eight different related subunits, each composed of eight separate gene products: CCTα, CCTβ, CCTγ, CCTδ, CCT𝜀, CCTζ, CCTη, and CCT𝜃 (Hynes et al., 1995; Liou and Willison, 1997; Gutsche et al., 1999; Bourke et al., 2002). The primary substrates for TCP-1 are cytoskeletal proteins, such as actin (Sternlicht et al., 1993) and tubulin (Yaffe et al., 1992; Sternlicht et al., 1993). The cytoplasmic chaperonin containing TCP-1 is necessary during the process of forming the native conformation of cytoskeletal protein polypeptide chains, in the case of α-tubulin and β-tubulin, and additional protein cofactors (Melki et al., 1997).

The actin and microtubule cytoskeleton is an indispensable compositions of host cells, which plays an important role in the attachment, internalization, endocytosis, nuclear targeting, transcription, replication, assembly and exocytosis of every virus, and the transport of progeny subviral particles (Radtke et al., 2006; Volkman, 2007; Mu et al., 2016). In addition, there exist a plethora of evidence showing that TCP-1 is involved in viral infection. Inoue et al. (2011) reported that chaperonin TRiC/CCT participates in the replication of the hepatitis C viral genome via interaction with the viral NS5B protein. Coghlin et al. (2006) reported that an active TCP-1 is potentially involved in the immune response against viral infection in *M. rosenbergii* (Arockiaraj et al., 2012). In our previous comparative subcellular proteomics analysis of different resistant silkworm strains following BmNPV infection, we found that BmTCP-1 showed an marked downregulation of expression in the midgut of resistant strains following BmNPV infection (Wang et al., 2017).

To the best of our knowledge, there is no relevant report available to date on TCP-1 in silkworm. To clarify the function of *BmTCP-1β*, the expression profiles of *BmTCP-1β* at different developmental stages and in various tissues were analyzed using RT-qPCR. To further define the role of *BmTCP-1β* in BmNPV infection, the alteration of BmNPV infection in BmN cells and the expression patterns of actin and tubulin were analyzed following knockdown and overexpression of *BmTCP-1β* using siRNA and the insect pIZT/V5-His-mCherry vector.

## Materials and Methods

### Silkworm and Virus

The silkworm strain, p50 (‘Dazao’), was maintained in the Key Laboratory of Silkworm and Mulberry Genetic Improvement, Ministry of Agriculture, Sericultural Research Institute, Chinese Academy of Agricultural Sciences, Zhenjiang, China. The first three instar larvae were reared on a fresh artificial diet at 26 ± 1°C, 75 ± 5% relative humidity, and a 12-h day/night cycle. The rearing temperature for the last two instars was reduced to 24 ± 1°C, but the other conditions remained unchanged.

Budded virus containing EGFP-tagged (BV-EGFP) BmNPV were kindly provided by Associate Professor Xudong Tang in School of biotechnology. Polyhedrin promoter was used, the EGFP was inserted between *BamH*I and *Xho*I, which is not fused with any protein. The amount of BV-EGFP (pfu/mL) was determined by the standard curve method. Briefly, vp39 of BmNPV was selected to constructed standard plasmid that was used to build a standard curve using RT-qPCR. The equal volume solution containing BV-EGFP (1 × 10^8^ pfu/mL) was added into the culture medium to study the infection process of BmNPV in different cell lines.

### BmN Cell Culture and Transfection

The silkworm ovarian cell line, BmN, was cultured in TC-100 medium supplemented with 10% (v/v) FBS, 200 μg/mL penicillin, and 100 μg/mL streptomycin at 28°C. Transfection was performed using Neofect^TM^ DNA transfection reagent (NEOFECT, Chian), according to the manufacturer’s instructions.

Cellular fluorescence images were taken using a Leica inverted research grade microscope DMi3000B camera and processed with the Leica Application Suite V4.6 software.

### Bioinformatics Analysis

The cDNA and deduced protein sequence of BmTCP-1β were analyzed using DNAMAN 8.0 (Lynnon Corporation, Quebec, Canada). The conserved motif was predicted on the SMART server^1^. The signal peptide was predicted by SignalP 4.1^2^. Sequences of orthologs were analyzed using the BLASTP tool^3^. The amino acid sequences of different species were aligned using the MUSCLE module of the MEGA6 software. A neighbor-joining tree was generated with a bootstrap of 1000 replications using MEGA6, the best DNA/Protein models of LG+G was adopt.

In the present study, STRING^4^ was adopted to analyze the protein-protein interactions (PPIs) of BmTCP-1β with cytoskeletal proteins. Due to the lack of proteomics information on *B. mori* in STRING, the PPI network was built using the database of another well-studied insect, *Drosophila melanogaster* (*D. melanogaster*).

### RNA Isolation and cDNA Synthesis

Total RNA was extracted with TRIzol Reagent (Invitrogen, United States) according to the manufacturer’s instructions. A NanoDrop 2000 spectrophotometer (Thermo Scientific, United States) was used to quantitate the RNA concentration. The purity of all RNA samples was assessed at absorbance ratios of A_260/280_ and A_260/230_, and the integrity of the RNA was confirmed by 1% agarose gel electrophoresis. The first strand of cDNA was synthesized using the PrimeScript^TM^ RT Reagent Kit with gDNA Eraser (TaKaRa, Japan), according to the manufacturer’s instructions.

### Quantitative Reverse Transcription PCR (RT-qPCR)

To analyze the transcriptional levels of *BmTCP-1β*, *actin* and *tubulin*, quantitative reverse transcription PCR (RT-qPCR) was adopted. All primers are listed in Table 1. RT-qPCR reactions were prepared with the SYBR Premix Ex Taq^TM^ Kit (TaKaRa) following the manufacturer’s instructions. Reactions were carried out using the QuantStudio^TM^ Real-Time System (Thermo Fisher Scientific, United States). The thermal cycling profile consisted of an initial denaturation at 95°C for 5 min, 40 cycles at 95°C for 5 s, and 60°C for 31 s. All reactions were performed in triplicate. Relative expression levels were calculated using the 2^-ΔΔCt^ method following the protocol described by Livak and Schmittgen (2001). In the present study, the *B. mori* glycerol-3-phosphate dehydrogenase-1 (*BmGAPDH*) gene was used as a reference standard (Guo et al., 2016). Statistical analysis was conducted using the SPSS software (IBM, www.ibm.com). One-way ANOVA with Tukey’s posttest was used to analyze the acquired data. The *P*-value < 0.05 was believed to be statistically significant.

**Table 1:** Primers used in RT-qPCR.

Gene ID	Forward Primer (5′–3′)	Reverse primer (5′–3′)
*BmTCP-1β*	TGGGTGTGACCGAGTCGTATG	GGGTGCTGCCTTCAGAATGTT
*Bmactin*	ATCCTCCGTCTGGACTTGGC	GTCACGAACGATTTCCCTCTCA
*Bmtubulin*	GCAACACGACAGCCATCCAG	TCGAAAGTGAGTTACAAAGGT
*vp39*	CAACTTTTTGCGAAACGACTT	GGCTACACCTCCACTTGCTT
*BmGAPDH*	CGATTCAACATTCCAGAGCA	GAACACCATAGCAAGCACGAC
*BmTCP-1β FD*	GGGGTACCATGATAGGAGATTTGGTAAAGAGTACTT	GGAATTCGGGTGCTGCCTTCAGAAT


**Table 2:** Primers used to synthesize siRNA.

Primer names	Sequence (5′–3′)
BmTCP-1β-1 Olig-1	GATCACTAATACGACTCACTATAGGGtgatggtgctacgatattgTT
BmTCP-1β-1 Olig-2	AAcaatatcgtagcaccatcaCCCTATAGTGAGTCGTATTAGTGATC
BmTCP-1β-1 Olig-3	AAtgatggtgctacgatattgCCCTATAGTGAGTCGTATTAGTGATC
BmTCP-1β-1 Olig-4	GATCACTAATACGACTCACTATAGGGcaatatcgtagcaccatcaTT
BmTCP-1β-2 Olig-1	GATCACTAATACGACTCACTATAGGGaagttggtgtacatcaaccTT
BmTCP-1β-2 Olig-2	AAggttgatgtacaccaacttCCCTATAGTGAGTCGTATTAGTGATC
BmTCP-1β-2 Olig-3	AAaagttggtgtacatcaaccCCCTATAGTGAGTCGTATTAGTGATC
BmTCP-1β-2 Olig-4	GATCACTAATACGACTCACTATAGGGggttgatgtacaccaacttTT


### siRNA-Mediated Knockdown of *BmTCP-1β* in BmN Cells

To knock down *BmTCP-1β in vitro*, two special targets of this gene’s functional domain were selected. The siRNA oligos were synthesized by Sangon Biotechnology, China (Table 2). The synthesis of siRNA was performed using the *in vitro* Transcription T7 Kit (for siRNA Synthesis) (TaKaRa, Japan), according to the manufacturer’s instructions. The concentration of siRNA was obtained using a NanoDrop 2000 spectrophotometer. The RNA quality was examined by agarose gel electrophoresis and subsequently stored at -80°C until use.

BmN cells (1 × 10^6^ cells/well) were seeded on to Costar 6-well cell culture clusters and cultured overnight at 28°C. Transfection was performed using Neofect^TM^ DNA transfection reagent (NEOFECT, China), according to the manufacturer’s instructions. Briefly, 4 μg siRNA oligo was added to 0.2 mL serum-free TC-100 media and mixed gently. Subsequently, 4 μL transfection reagent was added to the mixture of TC-100 and siRNA oligo, mixed gently, and incubated for 15–30 min at room temperature (RT). The mixture of siRNA and transfection reagent was then added to the culture medium, mixed gently, and the cells were cultured at 28°C. Each transfection was repeated three times. After a 24-h, 48-h and 72-h incubation with siRNA, BmN cells were harvested to extract the RNA. The RNAi efficiency of *BmTCP-1β* was assayed by RT-qPCR.

### Overexpression of *BmTCP-1β* in BmN Cells

The functional domain of *BmTCP-1β* was amplified with primers *BmTCP-1β FD* (Table 1, the underlined portions indicate the *Kpn* I and *EcoR*I restriction sites, respectively). The cDNA of p50 midgut was used to amplify the functional domain of *BmTCP-1β*. Purified PCR products were ligated into pMD-19T for sequencing. The functional domain of *BmTCP-1β* was obtained by digestion from the recombinant plasmid using *Kpn*I and *EcoR*I, and subsequent ligation into the pIZT/V5-His-mCherry plasmid to construct the transient expression vector pIZT/V5-His-mCherry-BmTCP-1β. Transfection was performed as described above.

## Results

### Characterization of the *BmTCP-1β* Sequence

The full-length *BmTCP-1β* cDNA (GenBank accession number: NM_001046644.1) consists of a 97 bp 5′-untranslated region (5′-UTR), a 928 bp 3′-UTR and a 1,611 bp open reading frame (ORF) encoding a 536 amino acid protein (Supplementary Figure S1). The theoretical MW and *p*I are 57.59 kDa and 6.32, respectively. BmTCP-1β has one functional domain, Cpn60 TCP-1, which was consisted of several members of HSP60 chaperone family and TCP-1 (T-complex protein) family.

BLASTP search showed that the BmTCP-1β amino acid sequence is most similar to that of *Spodoptera litura*, *Heliothis virescens* and *Amyelois transitella* TCP-1 (94% identity), *Helicoverpa armigera*, *Danaus plexippus*, *Papilio xuthus*, *Papilio machaon* and *Papilio xuthus* (93% identity), *Pieris rapae*, *Bicyclus anynana* and *Papilio polytes* (92% identity), followed by *Aedes aegypti* (78% identity) and *Aedes albopictus* (77% identity). Therefore, the high conservation of TCP-1β in different species shows that BmTCP-1β may serve as a molecular chaperone during protein folding in silkworm (Supplementary Figure S2).

The entire amino acid sequence of BmTCP-1β and those of other species were used to determine the evolutionary relationships among TCP-1β in different species. A phylogenetic tree consisting of BmTCP-1β and 17 other homologs was constructed. These genes were clearly classified into four groups: Lepidoptera, Hemiptera, Capparidales, and Rodentia. BmTCP-1β and its homologs from 12 other insects including *H. armigera*, *H. virescens*, *S. litura*, *D. plexippus*, *A. transitella*, *P. xylostella*, *B. anynana*, *P. machaon*, *P. polytes*, *P. xuthus*, and *P. rapae* were clustered into the Lepidoptera group (Supplementary Figure S3). *M. musculus* and *Arabidopsis* sequences shared a lower sequence identity with BmTCP-1β, indicating that the gene in their ancestors may have been diverged before the emergence of these orders.

**FIGURE 1:**
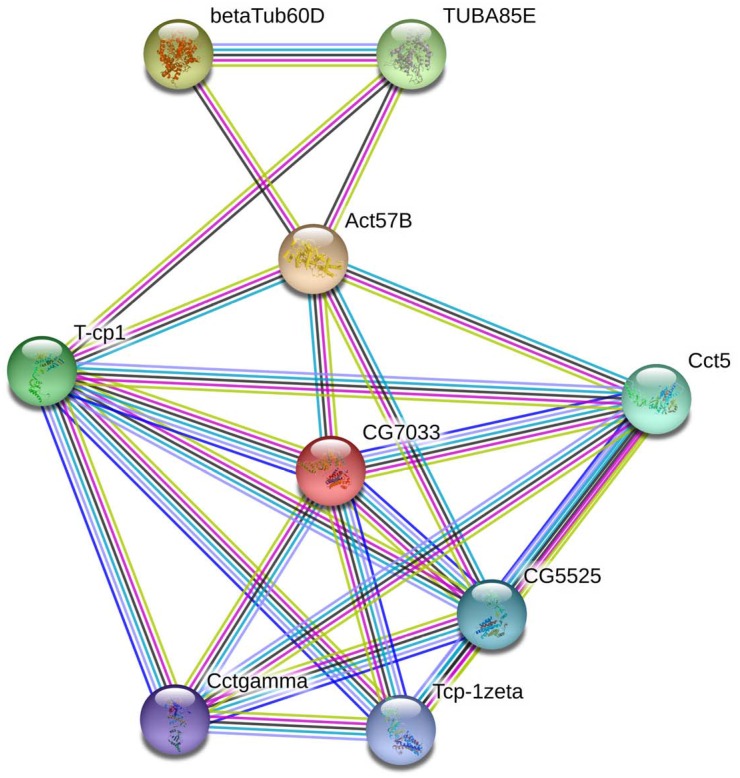
The interaction network of TCP-1β with selected cytoskeletal proteins was constructed based on the STRING website information using the database of another well-studied insect, *D. melanogaster*. CG7033, TCP-1β; Act57B, actin; β-Tub60D, β-tubulin; TUBAB5E, α-tubulin.

In living cells, many proteins can interact with each other, and these interacting proteins are expected to be involved in the same biological process or to function in the same subcellular compartment, which is supported by evidence that proteins in the same pathway are more interconnected (Barabasi et al., 2011). STRING contains abundant resources on physical and functional interactions and collects information from numerous sources, including experimental repositories, computational prediction methods, and public text collections (Jensen et al., 2009). To further investigate the relationship between BmTCP-1β and the selected cytoskeletal proteins, Actin and Tubulin, the functional association of these proteins was analyzed using the STRING 9.1 online software. A combined score was assigned to every protein-protein association pair in the software. This score was computed by combining the probabilities from several pieces of evidence and correcting for the probability of randomly observing an interaction. As illustrated in Figure 1, TCP-1β has a close relationship with actin and tubulin, especially actin, which suggests that BmTCP-1β may be involved in BmNPV infection by mediating the expression of cytoskeletal proteins.

### The Spatio-Temporal Expression Pattern of *BmTCP-1β*

**FIGURE 2:**
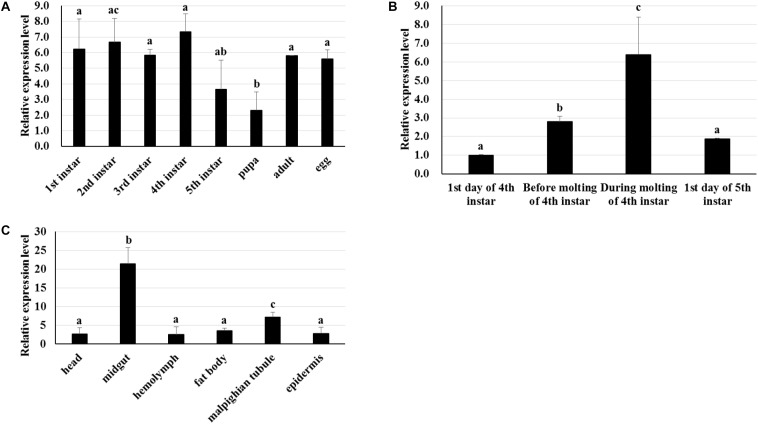
The spatiotemporal expression analysis of *BmTCP-1β*. **(A–C)** show the expression patterns of *BmTCP-1β* at different developmental stages **(A)**, during molting **(B)**, and in different tissues **(C)**. The data were normalized using *BmGAPDH* and are represented as the mean ± standard error of the mean, from three independent experiments. Relative expression levels were calculated using the 2^-ΔΔCt^ method. Statistical analysis was conducted using the SPSS software. One-way ANOVA with Tukey’s posttest was used. Significant differences are indicated by different letters, e.g., a, b, and c (*p* < 0.05).

RT-qPCR was used to analyze the transcriptome patterns of *BmTCP-1β* in different tissues and at various developmental stages. The total RNA of different tissues and developmental stages was extracted from the whole body, and agarose gel electrophoresis was used to assess the integrity of total RNA. The quality of cDNA was assessed using *BmGAPDH*. We found that the expression pattern of *BmTCP-1β* was not significantly different among various different developmental stages (Figure 2A). Additionally, *BmTCP-1β* showed relatively a higher expression level before the molting stage of fourth instar, and reached the highest expression level during the molting stage of fourth instar, while other selected stages showed significantly lower expression levels (Figure 2B). Moreover, *BmTCP-1β* showed significant specificity in tissue expression, with the lowest expression level observed in the hemolymph and the highest in the midgut (Figure 2C).

### Alteration Analysis of Selected Cytoskeletal Genes Following Knockdown of *BmTCP-1β in vitro*

**FIGURE 3:**
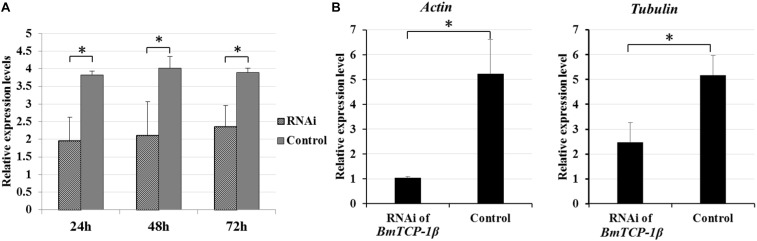
Expression analysis of selected cytoskeletal genes following addition of *BmTCP-1β* RNAi. **(A)**, expression analysis of *BmTCP-1β* following siRNA interference using RT-qPCR at different times. **(B)**, expression analysis of selected cytoskeletal genes using RT-qPCR. The data were normalized using *BmGAPDH* and are represented as the mean ± standard error of the mean, from three independent experiments. Relative expression levels were calculated using the 2^-ΔΔCt^ method. Statistical analysis was conducted using the SPSS software. One-way ANOVA with Tukey’s posttest was used. Significant differences are indicated by asterisks (*p* < 0.05).

To identify the best interference time of siRNA in *BmTCP-1β* expression, BmN cells were transfected with siRNA for three different time points, the control group was treated with transfection reagent without siRNA. The relative expression level of *BmTCP-1β* showed significant downregulation following siRNA treatment for 24 h, 48 h and 72 h. The significant downregulation of *BmTCP-1β* was initially 24 h following siRNA transfection; thus, an interference time of 24 h was selected for further analyses (Figure 3A).

To detect the connection of *BmTCP-1β* to selected cytoskeletal genes, RT-qPCR was adopted to analyze the expression patterns of *actin* and *tubulin* following knockdown of *BmTCP-1β*. The results show that *actin* and *tubulin* were both significantly down-regulated following addition of *BmTCP-1β* RNAi (Figure 3B), indicating that *BmTCP-1β* has a close relationship with *actin* and *tubulin*.

### Alteration Analysis of BmNPV Infection Following Addition of *BmTCP-1β* RNAi at Different Times *in vitro*

**FIGURE 4:**
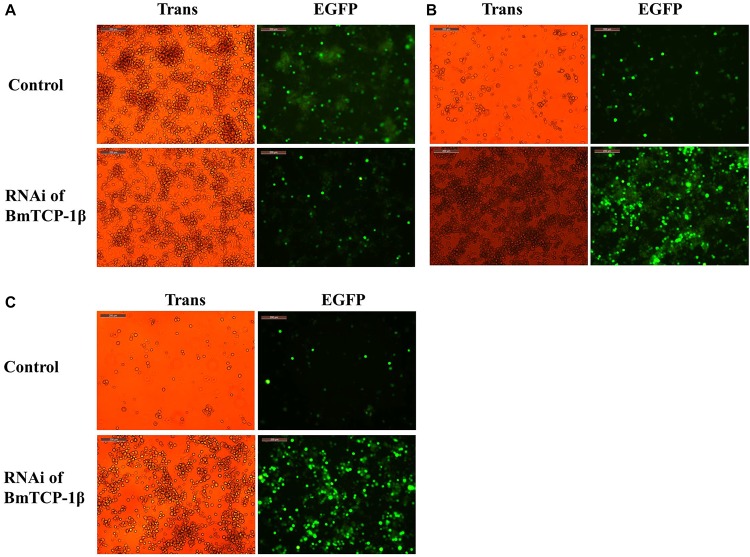
Infection analysis of BmNPV in BmN cells following addition of *BmTCP-1β* RNAi for different time periods. **(A)** 48 h after BV-EGFP infection. **(B)** 72 h after BV-EGFP infection. **(C)** 96 h after BV-EGFP infection. Scale bar, 200 μm. Trans (white), optical transmission; EGFP (Green), expressed following the replication of BV.

To identify the function of *BmTCP-1β* in the process of BmNPV infection, BmN cells infected with BV-EGFP were analyzed at different time points following knockdown of *BmTCP-1β* by siRNA at 24 h. A significant infection signal of BV-EGFP was found at 48 h post-infection, where the fluorescence signals of BmNPV in the control were significantly higher than those in the *BmTCP-1β* RNAi group (Figure 4A), indicating that *BmTCP-1β* promoted the infection of BmNPV. At 72 h post-infection, most BmN cells were dissociated in the control as compared with *BmTCP-1β* RNAi group (Figure 4B). At 96 h post-infection, there were only few of EGFP signals in the control group, while BmN cells treated with siRNA retained normal cell morphology (Figure 4C), which is consistent with the results obtained at 48 and 72 h, further confirming the vital role of *BmTCP-1β* in BmNPV infection.

### Overexpression of *BmTCP-1β* Induces Upregulated Expression of Selected Cytoskeletal Genes *in vitro*

**FIGURE 5:**
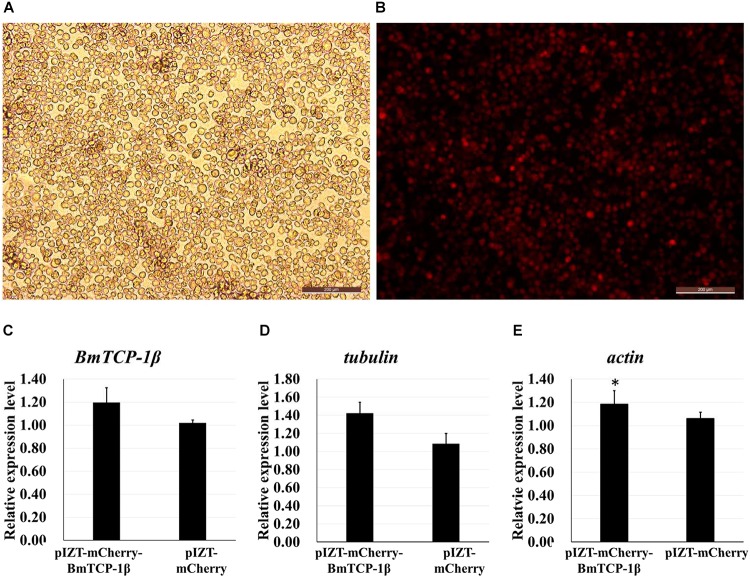
Correlation analysis of *BmTCP-1β* with selected cytoskeletal genes by overexpression of *BmTCP-1β* *in vitro*. **(A,B)**, overexpression of BmTCP-1β in BmN using the pIZT/V5-His-mCherry vector, scale bar = 200 μm. **(A)** Trans (white), optical transmission. **(B)** Transfection of BmN cells with the pIZT/V5-His-mCherry-BmTCP-1β vector, mCherry (red). **(C–E)** expression analysis of *BmTCP-1β*, *actin*, and *tubulin* using RT-qPCR. One-way ANOVA with Tukey’s posttest was used. Data were analyzed as described above. Significant differences are indicated by asterisks (*p* < 0.05).

The insect expression vector pIZT/V5-His-mCherry was used to overexpress *BmTCP-1β* *in vitro*. The purified PCR product of *BmTCP-1β* functional domain was inserted into the pIZT/V5-His-mCherry vector using *Kpn*I and *EcoR*I restriction enzymes. BmN cells were transfected with the recombinant bacmid to overexpress the BmTCP-1β protein, the stable cell line of pIZT/V5-His-mCherry-BmTCP-1β was screened by final concentration of 200ng/uL zeocin. The red fluorescence protein of mCherry indicates that BmTCP-1β was successfully overexpressed in BmN cells (Figure 5A,B), which was validated by RT-qPCR (Figure 5C). The expression levels of selected cytoskeletal genes in transgenic BmN cells were compared with those in wild-type BmN cells by RT-qPCR. The results show that overexpression of BmTCP-1β could significantly induce up-regulation of *actin* and *tubulin* *in vitro* (Figure 5D,E), which is consistent with the RNAi results.

### Overexpression of *BmTCP-1β* Accelerates the Infection of BmNPV in BmN Cells

**FIGURE 6:**
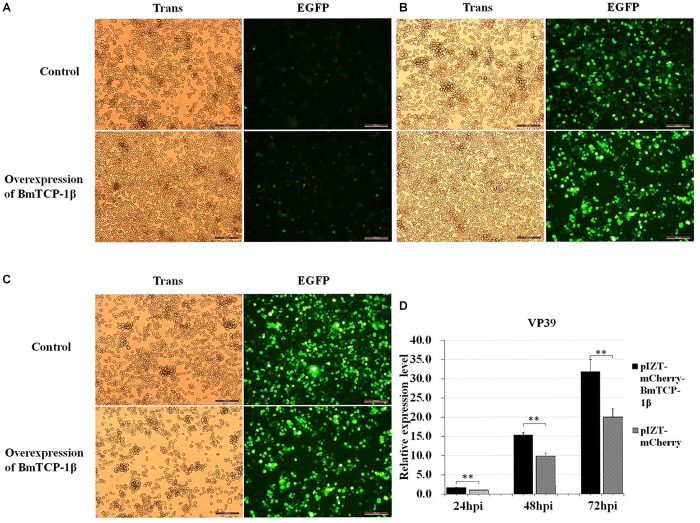
Infection analysis of BmNPV in wild type and transgenic BmN cells. **(A)** 24 h after BV-EGFP infection. **(B)** 48 h after BV-EGFP infection. **(C)** 72 h after BV-EGFP infection. Scale bar = 200 μm. **(D)** replication analysis of BmNPV following the overexpression of *BmTCP-1β*. The data was analyzed as described above. One-way ANOVA with Tukey’s posttest was used. Significant differences are indicated by asterisks (*p* < 0.05).

Based on the RNAi analysis, knockdown of *BmTCP-1β* delayed the infection of BmNPV in BmN cells. To further validate the function of *BmTCP-1β* in BmNPV infection, transcription of BV *vp39* gene in control and transgenic BmN cells at different infection times was analyzed following the overexpression of *BmTCP-1β* using an insect expression system. The results show that a significant infection signal was found 24 h post-infection, which was significantly higher in transgenic BmN cells than in control BmN cells (Figure 6A), consistent with the RNAi results. At 48 h post-infection, significant dissociation of BmN cells was found in the transgenic group as compared with control group (Figure 6B). At 72 h post-infection, most transgenic BmN cells were floating in the culture medium and numerous infected cells were dissociated, while the BmN cells in the control group retained complete cell morphology and were adhered (Figure 6C). Moreover, the number of BmNPV copies was detected using RT-qPCR, and the results show that the replication of BmNPV in the transgenic cells was significantly faster than that of in the control cells at all three selected time points (Figure 6D), which further validated the reasonable suggestion that the early infection signals in the transgenic cells, and *BmTCP-1β* accelerated BmNPV infection.

## Discussion

In our previous comparative subcellular proteomics analysis of different resistant silkworm midguts in response to BmNPV infection, many interesting proteins related to BmNPV infection were identified. Among these, we were interested in BmTCP-1β, which showed a significant downregulation following BmNPV infection, indicating that BmTCP-1β may be involved in BmNPV infection. TCP-1β in response to viral infection has been widely reported in other fields. Inoue et al. (2011) reported that a subunit of CTP1 could ligate to Negri bodies and promote rabies virus transcription and replication (Zhang et al., 2013, 2014), which is in agreement with our previous study reporting notable down-regulation of BmTCP-1β in the resistant strain, BC9, following BmNPV infection, but no differential expression in the susceptible strain, p50. Nevertheless, the underlying mechanism of BmTCP-1β in response to BmNPV infection remains unclear. In the present study, the molecular characterization and functional analysis of *BmTCP-1β* is described.

The biological information method was used to characterize the sequence of *BmTCP-1β*. The homologous alignment analysis shows *BmTCP-1β* shares a high similarity with its homologous genes in other species (Supplementary Figure S2), which was verified by phylogenetic analysis (Supplementary Figure S3), indicating its role in the process of protein folding. Moreover, BmTCP-1β has a close interaction with actin and tubulin on the STRING website using the *Drosophila* database (Figure 1), which is consistent with previous reports that TCP-1 is vital in protein folding to produce stable and functionally competent conformations (Blitvich et al., 2001; Olshina et al., 2016). The stable expression levels at different developmental stages indicate that *BmTCP-1β* is essential in silkworm development (Figure 2A). Furthermore, the relatively higher expression levels of *BmTCP-1β* during the molting stage suggests that the expression of *BmTCP-1β* may be regulated by molting hormones (Figure 2B). The highest expression level was found in the midgut, indicating its vital role in the process of BmNPV infection of midgut cells (Figure 2C).

Our previous proteomic data showed that BmTCP-1β may be involved in BmNPV infection. By combining the transcriptome data (Wang et al., 2016), we deduced that BmTCP-1β is involved in BmNPV infection by regulating the expression of cytoskeletal proteins. To validate this point, here, the expression of *BmTCP-1β* was knocked down using siRNA in BmN cells. The result showed that *BmTCP-1β* not only down-regulated the expression of selected cytoskeletal genes, including *actin* and *tubulin* (Figure 3), but also delayed the infection of BmNPV (Figure 4), indicating that our inference is reasonable. Moreover, to further validate the results of the *BmTCP-1β* RNAi described above, overexpression of *BmTCP-1β* was also carried out using the insect pIZT/V5-His-mCherry vector. The functional domain of *BmTCP-1β* was ligated into pIZT/V5-His-mCherry to overexpress BmTCP-1β in BmN cells (Figure 5). The results show that the expression levels of cytoskeletal genes were up-regulated in the transgenic cell lines and the infection time of BmNPV was also shorter than that in the control group (Figure 6). Based on the above analysis, it is reasonable to suggest that BmTCP-1β plays a vital role in the response to BmNPV infection by regulating the expression of cytoskeletal genes. Taken together, our work provides valuable data for the clarification of the underlying molecular mechanism of silkworm resistance to BmNPV infection.

**FIGURE 7:**
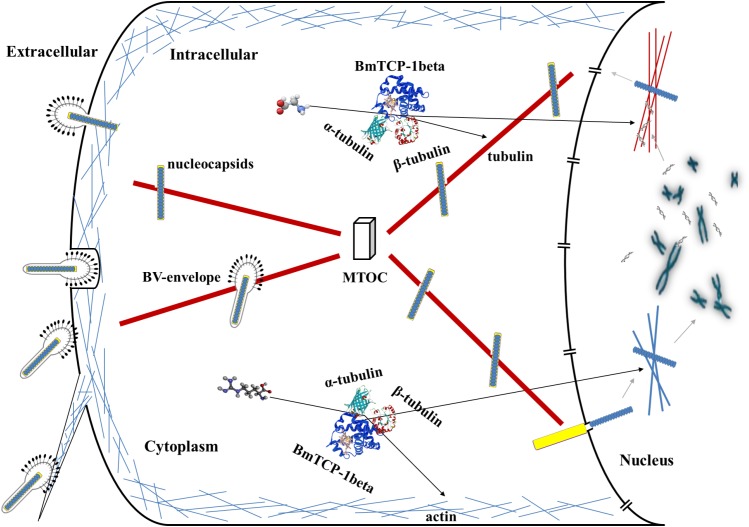
Hypothesized roles of BmTCP-1β in the response to BmNPV infection. MTOC, microtubule-organizing center.

Based on the above analysis in combination with a previous report about the role of the cytoskeleton in the process of baculoviral infection (Radtke et al., 2006), we hypothesize that BmTCP-1β plays a vital role in the process of BmNPV infection of host cells by regulating cytoskeletal proteins. The actin-mediated endocytic process is triggered by BV-envelope contact with the plasma membrane (Ascough, 2004) and surfing via filopodia toward an area with high endocytic activity (Pollard and Borisy, 2003), where it is internalized by endocytosis. Alternatively, BmNPV nucleocapsids can fuse with the plasma membrane. After traversing the actin cortex, either inside endocytic vesicles or by itself, free nucleocapsids or BV-envelopes inside vesicles are transported along tubulin toward the microtubule-organizing center (MTOC). From the MTOC, nucleocapsids are transported toward the nucleus, and upon binding to the nuclear pore, the nucleocapsids release their genome for replication (Radtke et al., 2006; Figure 7). The actin and tubulin involved in this process acquire their native confirmation in the presence of BmTCP-1β, and in the case of α-tubulin and β-tubulin, additional protein cofactors, which may explain the lower expression level of BmTCP-1β in the resistant strain, BC9, as compared with the susceptible strain, p50.

## Author Contributions

X-yW conceived and designed the experiments. X-yW, Z-mS, Q-yC, and XS performed the experiments. X-yW, Z-mS, and Q-yC analyzed the data. M-wL, Z-pX, and Y-cW contributed to reagents, materials, and analysis tools. X-yW, Z-mS, and J-pX wrote the manuscript.

## Conflict of Interest Statement

The authors declare that the research was conducted in the absence of any commercial or financial relationships that could be construed as a potential conflict of interest.

## Footnotes

1 http://smart.embl-heidelberg.de/

2 http://www.cbs.dtu.dk/services/SignalP/

3 http://www.ncbi.nlm.nih.gov/

4 http://string-db.org/
