# Supplementary material for: Knockdown of BmTCP-1β Delays BmNPV Infection in vitro
Source: Front Microbiol. 2019 Mar 22;10:578. doi: 10.3389/fmicb.2019.00578 (PMC6439466; doi:10.3389/fmicb.2019.00578)
Supplement: Supplementary file 1 [file Data_Sheet_1.pdf]

Supplementary Material

Knockdown of *BmTCP-1β* delays BmNPV infection *in vitro*

Xue-yang Wang[1,2#], Zuo-min Shao[1#], Qian-ying Chen[1], Jia-ping Xu[3], Xia Sun[1,2], Zhen-ping Xu[1,2], Mu-wang Li[1,2*], Yang-chun Wu[1,2*]

*** Correspondence:** Mu-wang Li, mwli@just.edu.cn; Yang-chun Wu, jkdwyc@163.com

1 Supplementary Data

Supplementary Material should be uploaded separately on submission. Please include any supplementary data, figures and/or tables. All supplementary files are deposited to FigShare for permanent storage and receive a DOI.

Supplementary material is not typeset so please ensure that all information is clearly presented, the appropriate caption is included in the file and not in the manuscript, and that the style conforms to the rest of the article.

2 Supplementary Figures

2.1 Supplementary Figures

```
1     ATGGTATCATTAAATCCAATTCGTATCCTTAAAAATGAAGCAGAAGAGGAGAAAGCTGAAGTTGCTCGGATGTCC
1      M  V  S  L  N  P  I  R  I  L  K  N  E  A  E  E  E  K  A  E  V  A  R  M  S
76    AGTTTCATCGGTGCTATTGCCATAGGAGATTTGGTAAAGAGTACTTTAGGTCCCAAAGGAATGGATAAGATCTTA
26     S  F  I  G  A  I  A  I  G  D  L  V  K  S  T  L  G  P  K  G  M  D  K  I  L
151   GTTTCCTGCGGGAGGAACTCTGGTCAGGTCGAAGTAACAAATGATGGTGCTACGATATTGAAATCTATCGGTGTG
51     V  S  C  G  R  N  S  G  Q  V  E  V  T  N  D  G  A  T  I  L  K  S  I  G  V
226   GATAATCCTGCTGCTAAAATCCTAGTAGATATGTCTAAGGTACAAGATGAAGAAGTCGGAGATGGCACCACATCT
76     D  N  P  A  A  K  I  L  V  D  M  S  K  V  Q  D  E  E  V  G  D  G  T  T  S
301   GTTACTGTTTTGGCTGCTGAATTACTTCGAGAGGCTGAAAAATTAATTGAACAAAAACTGCATCCTCAAACAATC
101    V  T  V  L  A  A  E  L  L  R  E  A  E  K  L  I  E  Q  K  L  H  P  Q  T  I
376   ATTGCTGGTTGGAGAATTGCTTCTGATGCAGCCAAACAAGCTCTTGCAGAAGCTAGCTTTGATCATCAGAAGAAC
126    I  A  G  W  R  I  A  S  D  A  A  K  Q  A  L  A  E  A  S  F  D  H  Q  K  N
451   TTAAATGAGGCTTCATTAAGAGTGGATTTAGAAAACATCGCACGGACCACACTGAGCTCAAAGATCCTTTCAAAT
151    L  N  E  A  S  L  R  V  D  L  E  N  I  A  R  T  T  L  S  S  K  I  L  S  N
526   CACAAAGAGCATTTCACAAAATTAGCAGTTGATGCAGTTTTGCGTTTGAAAGGATCTGGTAATTTAAAAGCTATT
176    H  K  E  H  F  T  K  L  A  V  D  A  V  L  R  L  K  G  S  G  N  L  K  A  I
601   CAAATTATCAAAATATCTGGAGGTCTTCTTGAAGAATCATTCTTGGATGAAGGGTTCTTGCTAAACAAAAAAGTT
201    Q  I  I  K  I  S  G  G  L  L  E  E  S  F  L  D  E  G  F  L  L  N  K  K  V
676   GGTGTACATCAACCAAAGAAAGTAGAAAATGCTAACATCCTTATTGCAAACACACCAATGGACACTGACAAGATT
226    G  V  H  Q  P  K  K  V  E  N  A  N  I  L  I  A  N  T  P  M  D  T  D  K  I
751   AAAGTGTTTGGTTCCACAATTAAAGTGGATTCAATGGCCAAGATTGCTGAATTGGAAGTAGCTGAGAAAGAGAAA
251    K  V  F  G  S  T  I  K  V  D  S  M  A  K  I  A  E  L  E  V  A  E  K  E  K
826   ATGAAGGACAAAGTGAACAAGATTTTAGCACACAAATGTAATGTCTTCATTAACAGACAACTTATATACAACTAC
276    M  K  D  K  V  N  K  I  L  A  H  K  C  N  V  F  I  N  R  Q  L  I  Y  N  Y
901   CCAGAGCAGTTGTTTGCTGATGCTGGAGTAATGGCAATTGAGCATGCTGATTTCGAAGGTATCGAAAGGCTTGGC
301    P  E  Q  L  F  A  D  A  G  V  M  A  I  E  H  A  D  F  E  G  I  E  R  L  G
976   TTGGTCACTGGAGGGGAAATTGTGTCAACATTTGACTCACCAGACAAAGTGAAACTTGGTCACTGCAAATTGATC
326    L  V  T  G  G  E  I  V  S  T  F  D  S  P  D  K  V  K  L  G  H  C  K  L  I
1051  GAAGAGGTTCTGATTGGTGATGAGAGCTTGATCCGCTTCTCTGGTGTAGCGTTGGGCTCGGCTTGCACGATCGTA
351    E  E  V  L  I  G  D  E  S  L  I  R  F  S  G  V  A  L  G  S  A  C  T  I  V
1126  ATCCGTGGTGCCACACAACAAGTTATCGACGAAGCTGAGCGTTCACTCCATGATGCGCTTTGCGTGCTTGCTGCT
376    I  R  G  A  T  Q  Q  V  I  D  E  A  E  R  S  L  H  D  A  L  C  V  L  A  A
1201  ACTGTAAAAGAACCGAAGGTCATATGTGGAGGAGGGGCTGGTGAAATGCTAATGGCAGAAGCAGCTTCCCGCGCC
401    T  V  K  E  P  K  V  I  C  G  G  G  A  G  E  M  L  M  A  E  A  A  S  R  A
1276  GGCGCTCGCACAGCCGGGAAGGAAGCCGCAGCAGCCGAAGCCTTTGCAGTCGCGCTCCGGAGACTGCCGTCCGCT
426    G  A  R  T  A  G  K  E  A  A  A  A  E  A  F  A  V  A  L  R  R  L  P  S  A
1351  GTCGCCGACAATGCTGGCTACGACAGTGCTGATCTCATTGCTCGACTCAGAGCTCATCATTCTCGAGGAGAAAAC
451    V  A  D  N  A  G  Y  D  S  A  D  L  I  A  R  L  R  A  H  H  S  R  G  E  N
1426  ACTATGGGATTAGATATGCAAAACGGCACTGTTGGTGATATGAAGAAGTTGGGTGTGACCGAGTCGTATGTCGTA
476    T  M  G  L  D  M  Q  N  G  T  V  G  D  M  K  K  L  G  V  T  E  S  Y  V  V
1501  AAGAGACAGGTCCTGTTGTCTGCGGCTGAAGCGGCCGAGATGATTCTTCGTGTCGATAACATTCTGAAGGCAGCA
501    K  R  Q  V  L  L  S  A  A  E  A  A  E  M  I  L  R  V  D  N  I  L  K  A  A
1576  CCCAGACGCCGCGGACCCGACCGTCGCCCATGTTAA
526    P  R  R  R  G  P  D  R  R  P  C  *
```

Supplementary Figure 1. The ORF nucleotide sequence of *BmTCP-1β* and its deduced amino acid sequence. The ORF of *BmTCP-1β* is 1611 bp encoding a 536 amino acid protein with a molecular weight of 57.59 kDa. The underlined protein shows the functional domain of BmTCP-1β, Cpn60 TCP-1.

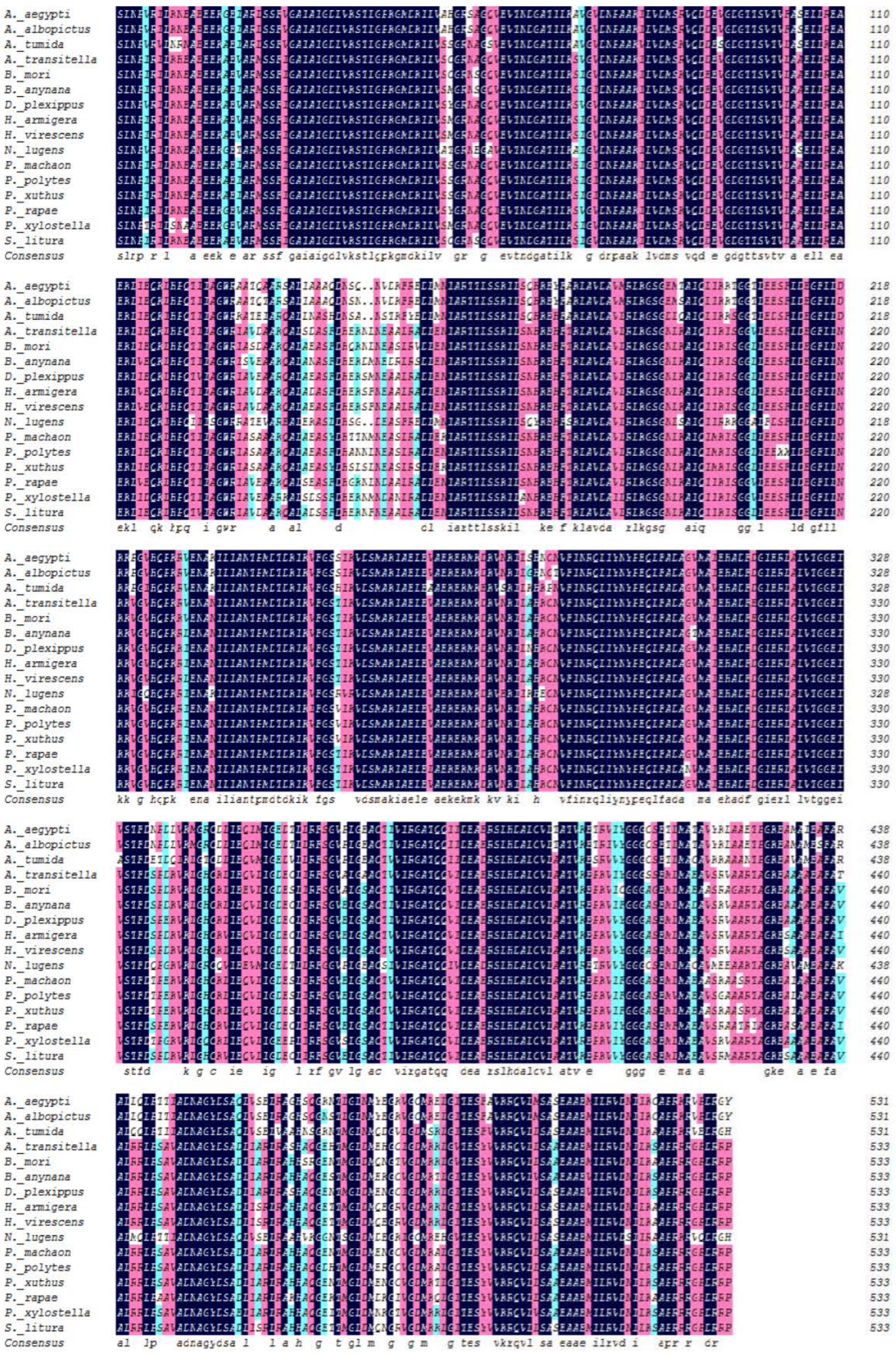

Supplementary Figure 2. Multiple sequence alignment of the BmTCP-1β ORF with its homologous proteins in other species. The deduced amino acid sequence of BmTCP-1β and its homologs in other species were retrieved from the NCBI database. Identical amino acids are highlighted in dark blue, and the positive amino acids are highlighted in pink and aqua.

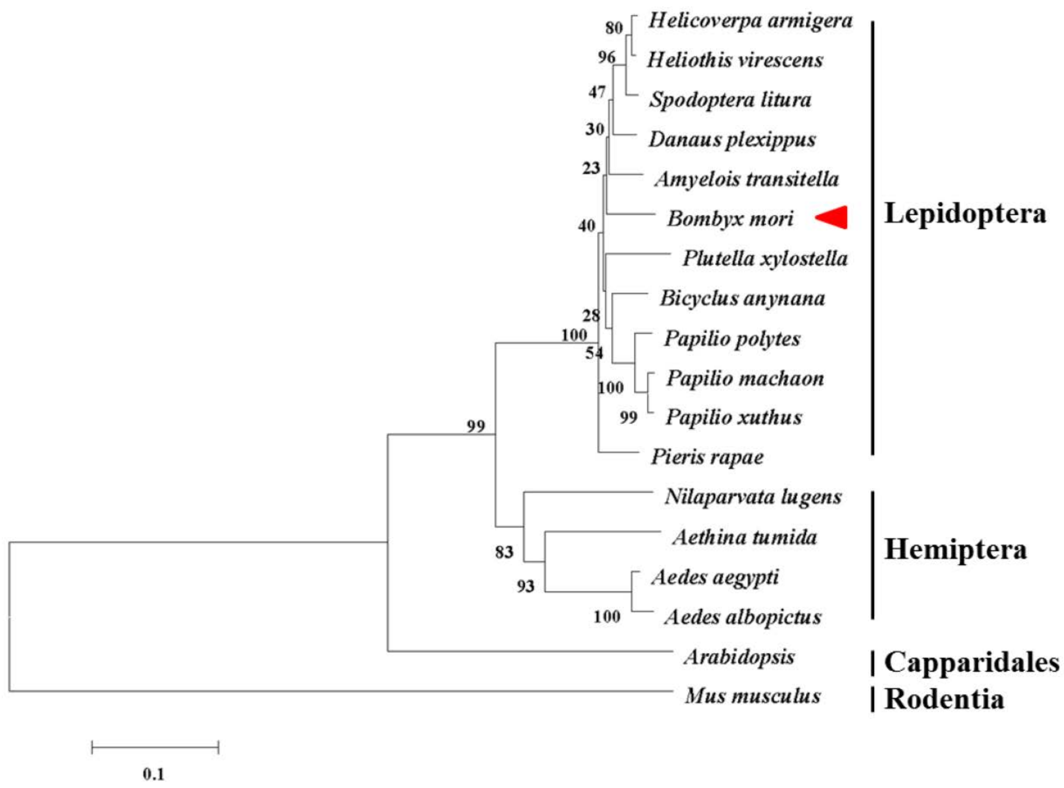

Supplementary Figure 3. Phylogenetic analysis of BmTCP-1β using the neighbor-joining method. The tree was constructed with pairwise deletion of gaps in MEGA 6.0. The percentages on the branches indicate bootstrap values from 1000 replicates. The tree is drawn to scale, with branch lengths in the same units as those of the evolutionary distances used to infer the phylogenetic tree. The evolutionary distances were computed using the p-distance method and are expressed as units of the number of amino acid differences per site. The analysis involved 18 amino acid sequences. All positions containing gaps and missing data were eliminated. Taxonomic names are shown on the right of the branches.
